# Recent Advances in Environment-Friendly Polyurethanes from Polyols Recovered from the Recycling and Renewable Resources: A Review

**DOI:** 10.3390/polym16131889

**Published:** 2024-07-02

**Authors:** Mengyuan Pu, Changqing Fang, Xing Zhou, Dong Wang, Yangyang Lin, Wanqing Lei, Lu Li

**Affiliations:** 1 School of Mechanical and Precision Instrument Engineering, Xi’an University of Technology, Xi’an 710048, China; pumengyuan37@163.com (M.P.); 18700919302@163.com (D.W.); 2 School of Printing, Packaging Engineering and Digital Media Technology, Xi’an University of Technology, Xi’an 710048, China; lyyang0506@163.com (Y.L.); lwq900529@163.com (W.L.); 3 Key Laboratory of Auxiliary Chemistry and Technology for Chemical Industry, Ministry of Education, Shaanxi University of Science and Technology, Xi’an 710021, China; lilu@sust.edu.cn; 4 Shaanxi Collaborative Innovation Center of Industrial Auxiliary Chemistry and Technology, Shaanxi University of Science and Technology, Xi’an 710021, China

**Keywords:** polyurethane, polyols, recycling, waste resource, bio-based materials

## Abstract

Polyurethane (PU) is among the most universal polymers and has been extensively applied in many fields, such as construction, machinery, furniture, clothing, textile, packaging and biomedicine. Traditionally, as the main starting materials for PU, polyols deeply depend on petroleum stock. From the perspective of recycling and environmental friendliness, advanced PU synthesis, using diversified resources as feedstocks, aims to develop versatile products with excellent properties to achieve the transformation from a fossil fuel-driven energy economy to renewable and sustainable ones. This review focuses on the recent development in the synthesis and modification of PU by extracting value-added monomers for polyols from waste polymers and natural bio-based polymers, such as the recycled waste polymers: polyethylene terephthalate (PET), PU and polycarbonate (PC); the biomaterials: vegetable oil, lignin, cashew nut shell liquid and plant straw; and biomacromolecules: polysaccharides and protein. To design these advanced polyurethane formulations, it is essential to understand the structure–property relationships of PU from recycling polyols. In a word, this bottom-up path provides a material recycling approach to PU design for printing and packaging, as well as biomedical, building and wearable electronics applications.

## 1. Introduction

**Figure 1:**
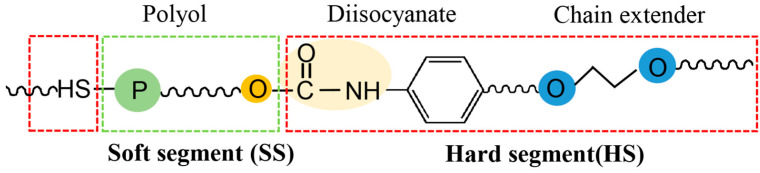
The chemical structure of PU.

PU, a kind of versatile polymer, is commonly produced by the synthesis of polyols and polyisocyanate through step-growth polymerization (Figure 1). PU, in the form of soft and hard foam plastics, elastomers, coatings, adhesives, films, hydrogels, waterproofing materials, paving materials and other forms, is widely used in many industries, such as leather, textile, printing and packaging, food processing, machinery, furniture, construction and even biomedical fields [1,2,3,4,5,6]. The soft segment of PU, composed of oligomer polyols, is mainly derived from petrochemical resources. However, with the crisis of energy shortage and environmental protection, the recycling of more sustainable resources as feedstocks is an excellent choice. Recently, there are two general methods for preparing sustainable polyols: recycling waste polymers to valuable monomers for waste-based polyols [7,8,9]; and exploring biomass resources to make monomers for bio-based polyols [10,11,12].

Plastic products are ubiquitous in our daily life and agricultural production. When these products reach the end of their life or use, they will be discarded and become waste plastic. There is a growing interest in the chemical recycling of waste polymers to recover valuable materials [13]. At present, some waste polymers could be degraded into value-added polyols as the raw material for PU, such as PET [14,15], PU [16] and PC wastes [17]. Meanwhile, the development and utilization of renewable resources to extract bio-based polyols to create sustainable “green” PU materials has also become a new bright spot [18]. Bio-based PU has undergone vigorous development in recent years, due to it saving resources, causing less environmental pollution, and having easy accessibility, a low cost and biodegradability [19]. Several bio-renewable materials have been introduced as feedstocks for PU, such as vegetable oil, nut shell, plant straw, lignin, starch, terpene and rosin, etc. [3,20,21]. These waste-based and bio-based polyols as feedstock are then reacted with various diisocyanate to facilitate PU production [22].

In recent years, several excellent reviews have discussed the recycling of waste PU materials and the advanced applications of waste PU derived materials in different fields [23,24,25]. However, recent advances in the preparation of PU materials from the extraction of valuable components from various recyclable resources have not been reviewed in detail. Additionally, some recent reviews have focused on the synthesis of bio-based PU, especially vegetable oil [26,27,28], and few of them have highlighted the waste-based polyol [29]. However, no review specifically covers the waste-based and bio-based PU as a whole. The challenge is how to produce PU with properties similar to traditional polyurethanes through effective resource conversion. Herein, we endeavor to focus on ever-expanding research of recyclable and environmentally friendly polyurethanes. The mechanism, types, synthesis, properties and applications of waste-based polyurethane and bio-based polyurethane were analyzed and compared with raw materials. Therefore, in this review we intended to aim at the most significant waste-based and bio-based PU and their products for sustainable printing and packaging, biomedical, buildings and wearable electronics applications. Finally, the cost and output of the waste-based and bio-based raw materials of PU are compared from a circular economy perspective. The true expansion of this circular economy market requires concerted action across the value chain.

## 2. Waste-Based Feedstocks for Polyurethanes

PU can be a kind of block polymer made from long and short chain raw materials. In general, long-chain polyols form soft segments, while hard segments are composed of short chain polyisocyanates [30]. Polyether, polyester, polycarbonate and other oligomers polyols can be used as soft segments [31]. The hard segments have a high glass transition temperature and melting temperature, providing the high hardness and strength of PU. Nevertheless, the soft segments have a lower glass transition temperature, giving PU flexibility, cross-linking ability and elasticity [32]. At the early stage, petroleum-based monomers were mainly used as precursors for polyols. Recently, there lies a huge potential in the development of new methods and technologies to produce polyols from waste polymers. Polymers are made up of a series of monomers reacted together in a polymerization process. The waste-based polymers can be classified based on their monomer sources [33,34], which have been key raw materials for fresh polymers. They can be chemical degraded to oligomer with carboxyl and hydroxyl end groups as building blocks to produce new polyester [35].

### 2.1. Waste Polyethylene Terephthalate for PU

Polyethylene terephthalate (PET), the main type of thermoplastic polyester, is one of the most multifunctional plastics prepared from terephthalic acid and ethylene glycol via polycondensation reaction nowadays [36,37]. PET is widely used to produce hollow containers in the packaging industry, such as soft drinking bottles in daily life. The number of global consumptions of plastic bottles currently is 1 million per minute, reaching 500 billion a year [38]. Most of these PET plastics are waste in landfills, thrown into incinerators or into rivers and oceans. So the recycling of plastic waste has become an urgent requirement of all sectors of society, and it is the urgent responsibility of the packaging industry [39]. There is an advanced technology that recycles plastic waste into raw materials. By chemical recycling, waste PET plastic bottles are extracted to polyester polyol, a key raw material for PU production, to enhance the commercial added value [40,41].

The main principle of the chemical recovery of PET is a depolymerization reaction, resulting in a polymer chain being broken up into oligomer polyols. Glycolysis [42,43,44,45], hydrolysis [46], alcoholysis [47,48] and biodegradation [49,50] are developing fast, and mainly applied on the scale of waste PET recycling to obtain high-value monomers and oligomers for the synthesis of PU (Table 1). In the following sections, we describe them separately.

#### 2.1.1. Glycolysis

**Figure 2:**
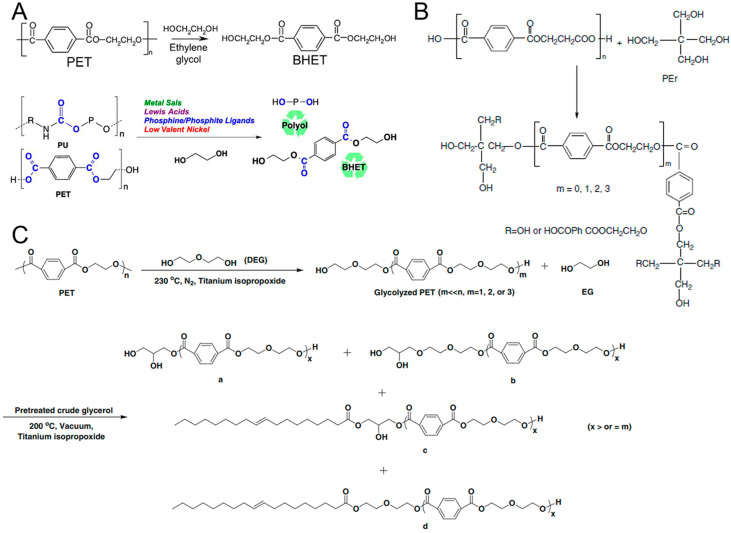
Glycolysis of PET. (**A**) The recovery of polyol from PU and BHET from PET plastic waste by transesterification using ethyleneglycol as a transesterification agent, metal salts, Lewis acids, phosphine/phosphite ligands with metal salts and low valent [Ni(COD)_2_] as catalytic precursors; reproduced with permission [51]. Copyright 2019, Elsevier. (**B**) PET depolymerization with Pentaerythritol (Per). Reproduced with permission [43]. Copyright 2013, Wiley. (**C**) Polyols were synthesized by glycolysis with polyester waste as raw material, diethylene glycol and crude glycerol as oligomer mixture (d), diols (c), triols (a,b). Reproduced with permission [42]. Copyright 2014, Springer.

Glycolysis, which is a reaction that converts an inhomogeneous system into a single phase, has been one of the most extensively prospective chemical recycling routes for most waste PET [51,52,53,54]. At the same time, it is the most commercially used route, and has been widely adopted by a number of well-known companies such as DuPont, Dow Chemical, Goodyear, Shell Polyester, Zimmer, Eastman, Kodak, and so on [55]. The glycolysis of PET is the transformation of PET to bis (hydroxyethyl) terephthalate (BHET) with the excess ethylene glycol as a transesterification agent, and the catalyst is mainly metal acetate (Figure 2A) [51,56]. This process essentially involves an esterification reaction through the breaking of the ester bond in the PET chain to produce BHET, dimer and oligomers [57].

In past and present, various oligomer polyols synthesized from waste PET plastic bottles via glycolysis are demonstrated along with their potential applications in Table 1. Atta et al. recycled PET waste to suitable polyol oligomers using trimethylolpropane or pentaerythritol for preparing PU foams (Figure 2B) [43]. The degradation of PET with ethylene glycol (EG) lead to the formation of hydroxyl groups in the resulting oligomers. Luo et al. reported that waste PET plastic bottles were degraded by diethylene glycol (DEG), producing monomeric or oligomeric diols with low molecular weight; then, PET degradation products, as raw materials, reacted with crude glycerol for producing polyols and PU foams (Figure 2C) [42]. Scremin et al. introduced the glycolysis reaction of PET using diethylene glycol, and the final glycolysis product PET polyol was used in PU adhesives by the one-shot method [52]. As can be seen, oligomers produced by glycolysis are mostly terminated by hydroxyl groups. In addition, Ramin Shamsi et al. reported that an efficient and reasonable green synthesis route was adopted, to add carbon nanotubes into polyurethane prepared from PET waste to obtain nanocomposites with excellent mechanical properties [58]. Meanwhile, the swelling properties of crosslinked polyurethanes in various non-polar and polar solvents were studied. The diffusion distribution and adsorption kinetics in different solvents were evaluated [59]. Therefore, the degradation product, polyols, can be applied into a valuable raw material for the later preparation of other polymer compounds.

#### 2.1.2. Hydrolysis

**Figure 3:**
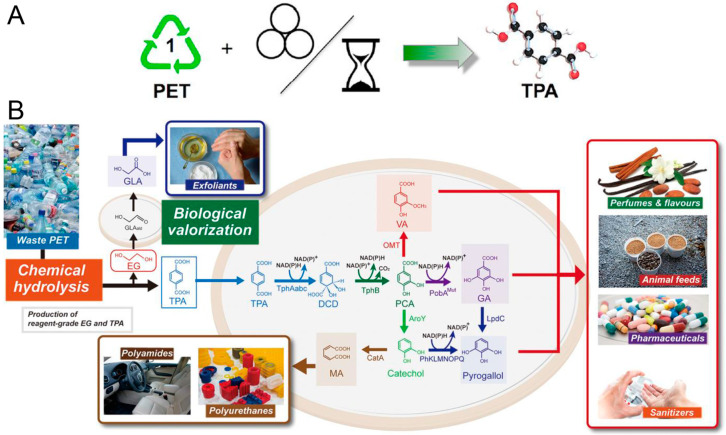
Hydrolysis of PET to TPA. (**A**) Alkaline solid-state PET hydrolysis into TPA by vapor-assisted aging under different relative humidity levels and solvent vapors. Reproduced with permission [62]. Copyright 2020, Wiley. (**B**) The overall plan of the upgrading and utilization of a waste PET biological refinery. Reproduced with permission [46]. Copyright 2019, American Chemical Society.

The hydrolysis of PET is performed so that the ester bond is attacked by water molecules under a certain temperature and pressure, producing terephthalic acid (TPA) and EG [60]. It is the reverse of the polycondensation reaction used to make the polymers in the first place, in which the addition of water or water vapor to the process causes decomposition [61]. Here are some new hydrolysis methods. Vjekoslav et al. investigated a convenient and quantitative hydrolysis method of waste PET into TPA by mechanochemical milling at ambient temperature and pressure (Figure 3A) [62]. Kang et al. developed a method using the microwave-assisted hydrolysis of PET into TPA by ZSM-5 zeolite catalyzed in neutral media [63]. TPA is extensively used in chemical building blocks for the synthesis of other chemicals, such as biodegradable plastics and the reproduction of PET by polymerization with EG, a characteristic of closed-loop recycling. Not only that, a new way to convert PET hydrolysis products to higher-value monomers, in which waste PET was decomposed into TPA and EG monomers and then the monomers were polymerized to aromatics and aromatic-derived compounds by serious microbial catalysts, is to be applied for manufacturing drug, cosmetics, disinfectant, animal feeds, bio-based monomers, and so on (Figure 3B) [46].

#### 2.1.3. Alcoholysis

**Figure 4:**
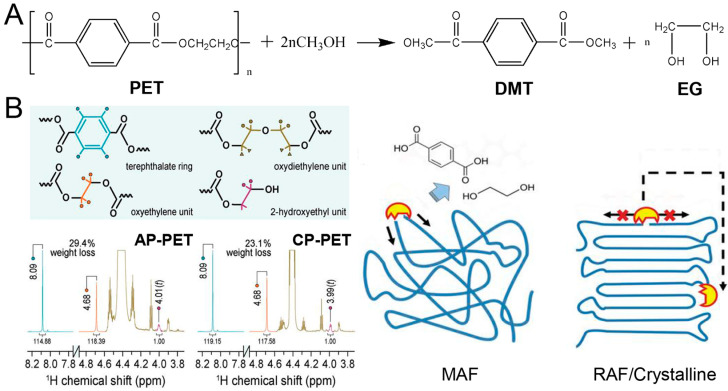
Alcoholysis of PET produces DMT. (**A**) The main depolymerization reaction of PET in supercritical methanol to DMT. Reproduced with permission [64]. Copyright 2015, Elsevier. (**B**) ^1^H spectra recorded by AP-PET and CP-PET in deuterated CDCl_3_/HFIP; The right figure shows PET degradation products were ethylene glycol and terephthalic acid, which indicated that the outer chain fracture occurred. Reproduced with permission [50]. Copyright 2019, Wiley.

Another effective method for the trans-esterification reaction of the waste PET with methanol is alcoholysis, to prepare the dimethyl terephthalate (DMT) and EG. DMT is a monomer of polyester, mainly applied for the synthesis of polyester. Liu et al. recycled waste PET to DMT and EG under a supercritical methanol, and researched the effect of alcoholysis temperature and time on yield (Figure 4A) [64]. Actually, DMT from alcoholysis products is recycled in the generation of polyester. Jadhav et al. researched that transesterification reaction of DMT and EG to prepare BHET, which is a precursor for PET [65]. Therefore, a closed-loop process for waste PET treatment and synthesis was formed. What’s more, it is a good solution for the maximum utilization of resources, and to release the pressure of waste pollution. Imek et al. found that waste PET depolymerized to dioctyl terephthalate by alcoholysis with isooctyl alcohol, which was applied in construction materials [47].

#### 2.1.4. Biodegradation

However, some new technologies appearing in recycling PET waste are biodegraded with bacteria. In 2016, Shosuke Yoshida et al. isolated a novel bacterium, which hydrolyzes PET to produce MHET, TPA and EG [49]. Sadeghi et al. introduced Bis (2-hydroxy ethylene) terephthalamide from PET to the ring-opening polymerization of Ɛ-caprolactone, and then synthesized a new biodegradable PU with HDI [66]. Wei et al. revealed that postconsumer PET food packaging containers were degraded by hydrolysis as promising biocatalysts, and the degradation production was more than 50% at 70 °C [50]. The nuclear magnetic resonance spectroscopy showed that the oxydiethylene unit, oxyethylene unit and 2-hydroxyethyl could form during the PET enzymatic degradation. The schematic illustration of the amorphous fraction of PET was degraded to ethylene glycol and terephthalic acid, indicating the exo-type chain scission (Figure 4B).

There are various PET degradation methods to meet the recycling treatment of different situations. By comparing the data presented in Table 1, it becomes evident that diverse degradation methods necessitate distinct conditions and yield dissimilar degradation products. The products are classified as follows: glycolysis converts PET to BHET, with ethylene glycol or other glycol, hydrolysis degrades PET to TPA under acidic or basic conditions, and the alcoholysis reaction depolymerizes PET to DMT using methanol. These resultant products can be employed for polyurethane preparation through various treatments to generate a wide range of polyurethane products. In particular, the glycolysis of PET involves the transesterification of hydroxyl compounds, resulting in reaction products with terminal hydroxyl groups that can be used to synthesize PU. These polyol products are then used as raw materials for the preparation of fresh polymer, such as PET, a characteristic of closed-loop recycling, as well as PU, used for producing various foams, coatings and adhesives [42,52,53].

### 2.2. Waste Polyurethane for PU

It is well known that PU is a block polymer synthesized from polyols and isocyanates, which are extremely valuable petrochemical materials. So, PU waste can also be recycled or recovered as a real value material in order to be put to further use. Because the strong polar carboxylate group and high density of hydrogen bonds between the molecules of PU is insoluble in non-polar groups, it possesses good oil resistance, chemical resistance, mechanical performance and wear resistance [67,68]. Owing to its excellent performance, it is widely used in many fields. As a consequence, a lot of PU waste exists in many forms: foams, films, elastomer and adhesive applied to cars [69], furniture [70], electrical appliances [71], buildings [72] and so on. From the direction of ecology, it is extremely easy for PU waste to contaminate the soil and destroy its components, as PU is difficult to degrade and its products, such as aromatic amines, are toxic [73].

On the other hand, PU feedstock recovery processes, to obtain high-value polyols as raw materials, are one of the most productive research topics today. The PU waste recovery methods include a series of physical and chemical processes, through which waste PU can either be cut into fragments which will be dissolved into fundamental hydrocarbon units, or depolymerized into constituent monomers as raw material to prepare new chemical products [16,74]. A series of research about the degradation of PU waste to obtain polyol performed by some reactions have been carried out. The recovery of the original raw materials, essentially the monomers of the polyols, can be applied in the synthesis of new PU products [75]. In order to understand the chemical recovery of PU waste in more detail, it is introduced in the following parts: hydrolysis, aminolysis, phosphorolysis and the hydroglycolysis of urethane and urea groups [76,77,78,79,80,81,82].

The main products of the hydrolysis reaction of polyether-based PU result are a diamine, a polyol and carbon dioxide [83]. The resulting polyol, with a similar composition to that of original polyether polyol, could replace the original polyols used for PU [84]. Furthermore, the resulting diamines are applied in isocyanates synthesis as the precursors, or given rigid characteristics by the alkoxylation of polyols or alkylene oxide. Additionally, due to the existence of ester groups, the polyester-based PU is hydrolyzed into monomers, including diacids and glycols or polyols [85]. Actually, the hydrolysis of polyester and polyether PU initially takes place in the hard segment through the decomposition of weak intermolecular hydrogen bonds between urethane groups on PU chains. The next step is the hydrolysis of the C=O group in the soft segment [86].

The glycolysis of the PU polymer is actually a transesterification reaction between carbamic ester and glycol, in which hydroxyl group of ethylene glycol replaces the ester group of urethane band [87,88,89,90,91]. A polyol mixture was composed of waste PU glycolysis products and aromatic polyols extracted from diphenylmethane diisocyanate, which could build up the physical and mechanical properties, heat resistance and fire resistance of rigid PU foams [92,93]. Shin et al. found that PU foam was made from recycled polyols with superior mechanical strength and thermal insulation performance compared with conventional foam [94]. Interestingly, glycolysis with polyfunctional alcohols can yield a mixture of biphasic products. It is composed of apolar polyether polyols in the upper phase, and aromatic compounds and polar glycolysis agents in the lower phase. Vanbergen et al. investigated the recovery of polyether polyols with high purity (97%) by fractional glycolysis in diglycerol, which could be used to replace the original polyether polyols in the later preparation of novel flexible PU foam materials [95]. Using split-phase glycolysis technology to recover PU production waste and product waste after consumption, closed-loop recycling can be realized. In addition, the secondary reaction is also unavoidable, resulting in various by-products, such as amines, isocyanates and unsaturation [96]. The main advantages of glycolysis are the low reaction temperature, short glycolysis time and the glycolysis product for producing new PU. This means it is also available for when these mixed polyol and isocyanate products could be reused and recycled as feedstock for the regeneration of PU with superior performance, and has been put into industrial production [87,97].

The aminolysis of PU is actually a chemical reaction, in which the ester group of urethane is exchanged with the amine group [16]. It takes place with dibutylamine, ethanolamine, lactam or lactam admixture and other amines react to break the chemical bond of PU waste under the high-temperature and high-pressure conditions [98]. The resulting decomposed polyol, the main products of ammonolysis, could be used as an alternative virgin polyol in fabricating PU. However, some researchers found that amine contaminants seriously affect the properties of polyols. Consequently, through the reaction of propylene oxide, the amine contaminants are separated from the recycled polyols, which can be applied to synthesize new PU with excellent performance [99,100]. In particular, aminolysis with monoethanolamine forms a hydroxyalkyl urea and an alcohol. Then, an intramolecular reaction occurs in hydroxyalkyl urea to form a cyclic urethane. Lastly, the reaction between the oxazolidone and monoethanolamine produce a bis(hydroxyethyl) urea as a chain extender in PU.

The phosphorolysis of polyurethanes is the ester exchange reaction processing with phosphoric and phosphonic acids, occurring between urethane and ester alkoxy group [101,102]. The process allowed the final products to mainly contain a mixture of oligomer of phosphorus and chlorine [103]. It can be used as a raw material of new PU, especially in the improvement of flame-retardant properties, adhesion properties and ultraviolet resistance [104,105]. Heinen et al. studied that the more phosphorus content in the oxygen index of PU foam, the greater the influence on the reduction in flammability [106].

Hydroglycolysis is a multiple method which is performed by combing water and glycols under a certain condition. PU reacts with diethylene glycol in an alkaline environment to form polyols and various intermediate chemicals [107]. The recovered polyols can also be resynthesized into PU products [108]. Unfortunately, the obtained polyol product is normally more complicated than conventional glycolysis. However, it is still a good way to deal with the complex mixture of dirty and contaminated PU waste that would otherwise have to go to landfills [109].

As can be seen from Table 2, most of the waste PU degradation products by different methods contain polyols. Therefore, the degradation products can be used as new raw materials to synthesize PU again. However, the products of waste PU after ammonolysis also include aromatic amine alkanolamine derivatives. These can be used to synthesize a variety of materials, including epoxy resins, polyesters, and polycarbonates. The PU waste must be treated and recycled effectively, not only to prevent pollution and protect environment, but also to reduce production cost and improve the resource utilization rate. More briefly, the pure monomer materials are obtained from waste PU through effective chemical treatment, and then these monomers are used as raw materials to prepare new products. Today, many companies exhibit their recovered polyols from waste in the chemicals market, compared with PU that has not been resynthesized.

### 2.3. Waste Polycarbonate for PU

Polycarbonate (PC) is an engineering plastic made from carbon dioxide (CO_2_) and epoxides, which has been widely used to prepare biocompatible biomaterials with environmental and economic benefits [110,111]. PC waste might be just like other waste, re-processed to recover raw materials by means of chemical recycling, such as methanolysis, hydrolysis, glycolysis and hydroglycolysis [112,113,114,115]. The carbonate group of PC could be completely converted to new phenol-containing carbamate derivatives, especially 2, 2-bis(4-hydroxyphenyl)propane (BPA) with mono- and di-hydroxyalkyl ethers, which are subsequently used as PC polyols in the synthesis of PU materials. Simultaneously, low molecular weight PC has been commercialized as a feedstock for the preparation of PU materials, such as elastomers, foams, adhesives, coatings [116,117,118,119,120,121].

**Figure 5:**
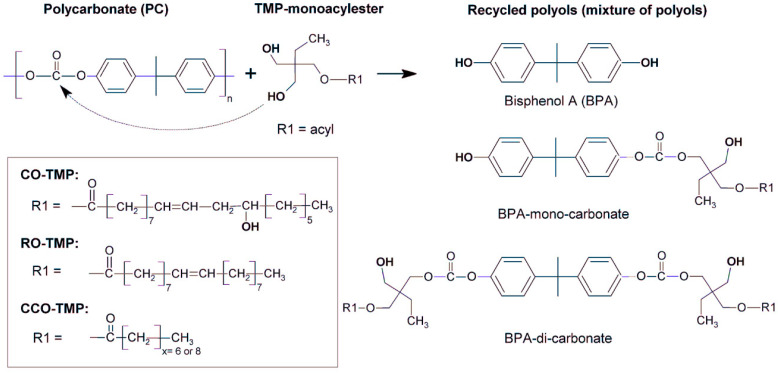
The reaction scheme of PC waste and transesterified oils (CO-TMP, RO-TMP and CCO-TMP) for recycled polyols. The inset shows the acyl structure of the TMP monoester present in transesterification oils. Reproduced with permission [122]. Copyright 2018, Tech Science Press.

Polycarbonate polyols show excellent chemical and thermal properties, especially better hydrolysis resistance, as well as the higher ageing resistance and biodegradability of PU products than polyester and polyether polyols in PU [17]. Beneš et al. successfully applied these to transform PC waste from scrap vehicles to valuable liquid polyols with hydroxyl number 250 mg KOH·g^−1^ by reacting them with transesterified oils (Figure 5) [122]. However, the PC recycling could be degraded into hydroxyl N,N′-diphenylene-isopylidenyl biscarbamates with low crystalline, resulting in low tensile properties. It was found that 2,2′-(ethyldioxy) diethylamine was added to the recovered PC monomer without any chemical cross-linking or blending reaction, and excellent thermoplastic properties of PU elastomers were obtained [117]. Additionally, recent studies demonstrated that polycarbonate diols in the soft segment of waterborne polyurethane (WPU) dispersions assured the excellent hydrolysis resistance and UV-curable ability. Moreover, it has been determined that polycarbonate PU is a hopeful strategy in biomedical field, such as interventional devices. Li et al. has found that novel phospholipid-based polycarbonate PU films could be coated on biomedical metal plates to build a bionic antifouling surface with excellent tensile properties [119].

With increasing attention paid to safety issues, there is a sustainable growth interest in developing green synthesis pathways for related polymers, such as non-isocyanate PUs (NIPUs). The synthesis of NIPUs using dicyclic carbonates prepared from carbon dioxide and epoxides has been widely studied [123,124]. Yang et al. proposed that an ideal route towards high molecular weight NIPUs was those synthesized by ammonolysis reaction with dicyclic carbonates from renewable resources [125].

To sum up, PET, PU and PC waste could be degraded into value-added polyols as the raw material for PU. This allows for the preparation of a rich variety of polyurethane product types, including foam, coating, and adhesive. The technology of preparing polyols from recycling resources makes this the leader in sustainable PU commercialization. The method can be a promising alternative to the traditional mechanical recovery of waste. Generally, since the raw materials applied in PU stem from fossil-based energy, there is a growing trend towards recyclable alternatives to petroleum. In this manner, the utilization of waste resources as chemical feedstocks for the chemical industry instead of oil is a sustainable alternative. This technology not only solves the solid-waste recycling issue, but serves as a source of raw stock in place of petroleum-based products, so there should be room for development in today’s world.

## 3. Bio-Based Feedstocks for Polyurethanes

Biomass resources, a kind of renewable energy, and its rational development and utilization, will effectively solve energy and ecological environment issues. With the rapid development of the industry, fossil resources tend to be exhausted gradually, so using low-cost biomass resources as raw materials meets the requirements of green development in both economic and environmental aspects. Polyols, an important component of PU, can be obtained from the biomass materials available, such as vegetable oil, cashew nut shell liquid, rosin, terpene and lignin [126]. Bio-based polyol was used as the soft segment and reacted with isocyanate to obtain the corresponding PU. These bio-based PUs have large potential in the polymer field and have excellent thermal-physical and mechanical properties, competing with traditional petroleum-based PUs. In particular, WPU merged as a new chemical candidate for ecofriendly adhesives and coatings has been greatly promising for bio-based applications. The content of this section is to review the process of bio-based PUs and modified PU by biomass materials.

### 3.1. PU from Renewable Monomers

#### 3.1.1. Vegetable Oil

**Figure 6:**
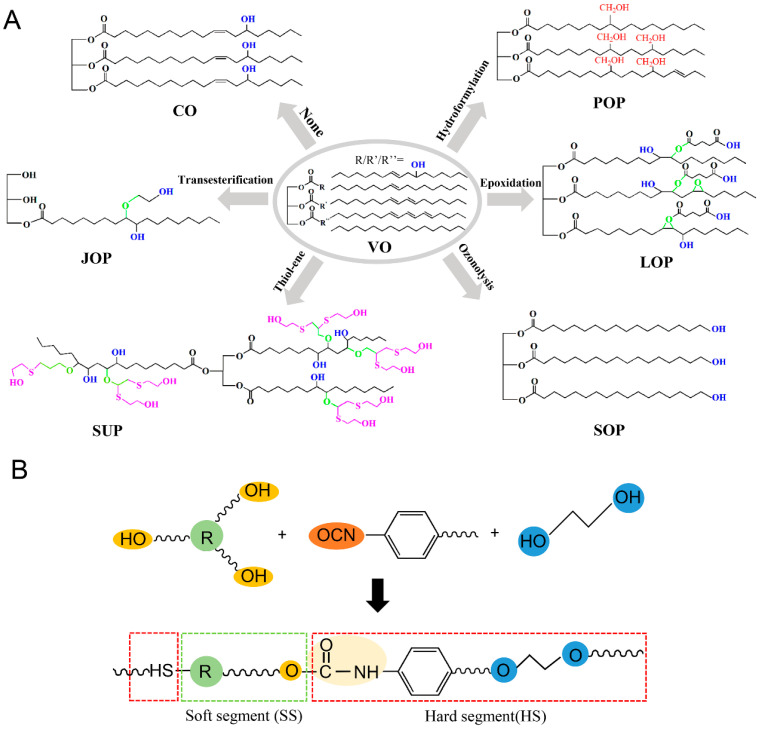
(**A**) The chemical composition of vegetable oil (VO)-based PU and the most common routes of VO-based polyols: castor oil (CO), palm oil-based polyol (POP), jatropha oil-based polyol (JOP), sunflower oil-based polyol (SUP), linseed oil-based polyol (LOP), soybean oil-based polyol (SOP). (**B**) The chemical structure (soft and hard segments) of polyurethane.

Vegetable oils (VOs) are one of the most abundant renewable resources, which hold great promise as raw materials to support the chemical conversion in a wide range. This can arise from the characteristic molecular structure, which mainly contains triglyceride, three long chain fatty acids, C=C bonds, as well as the actively terminal ester group, as illustrated in Figure 6A [127]. Thus, the desired structure can be obtained by chemical reactions between VOs as the soft segment (polyols) and isocyanate as the hard segment in PU, as depicted in Figure 6B. Some VOs have natural hydroxyl groups and can be directly used as polyols, such as castor oil. However, some VOs must be modified to introduce hydroxyl groups into the chains according to the C=C bonds and terminal ester group, such as soybean, sunflower, linseed oil, etc. [2]. Owing to the merits of green, inherent biodegradability and low cost, VOs have been employed to enhance the elasticity and mechanical properties of PU in industry [128] However, the demerits caused by the hydrophilic carboxylate groups in VOs-based PU should not be ignored, including low resistance for chemical agents and heat, and physicomechanical stability [129].

Castor oil (CO) can be directly employed as a polyol to react with isocyanates, which possesses active hydroxyl bonds (hydroxyl index ~163 mg KOH/g), illustrated in Figure 6A [130]. The three distributed active hydroxyl groups can facilitate the crosslinking of PU soft segment to obtain significant mechanical properties and thermal resistance [26]. It is reported that the unmodified castor oil contributes to the mechanical properties, and the water and chemical agent resistance of the WPU because of the existence of hydrophobic triglyceride structure [131,132]. In addition, the castor oil can also react with some diols, such as ethylene glycol (EG), glycerol, pentaerythritol, and sorbitol, to produce derivatives with molecular weights and different hydroxyl indexes.

All these derivatives are able to react with isocyanates to prepare WPU coatings [133]. Interestingly, it is expected that the derivatives from castor oil can be used as isocyanates to potentially prepare bio-based WPU [127]. As for the VOs without active hydroxyl groups, including soybean, sunflower, linseed, jatropha, palm oil and so on, it is necessary to introduce active hydroxyl groups into these VOs’ chains by chemical modification. Currently, five routes, including thiol-ene reaction, epoxidation, hydroformylation, ozonolysis and transesterification, have been developed to convert these VOs into PU polyols by decorating the C=C bonds or esters on their chains, as depicted in Figure 6A.

For example, soybean oil is commonly modified via thiol-ene reaction and epoxidation. The C=C bonds can be epoxidized to graft to the active hydroxyl groups, which can endow soybean oil multi-functional properties as bio-polyols, bio-emulsifier and even non-isocyanates agent for the preparation of PU [83]. Dodangeh et al. synthesized a bio-polyol by epoxidized soybean oil to prepare PU. The lap shear strength and tensile strength can be as high as 6.478 MPa and 7.173 MPa, respectively [134]. Moreover, the epoxidized soybean oil can also play the role of bio-based emulsifier with glutaric acid, which can be alternative for preparing WPU instead of typical petroleum-based emulsifiers [135]. As to the sunflower oil, it can be epoxidized to obtain active hydroxyl groups to prepare bio-polyols [136,137]. Owing to the carboxylic acid group on the chain, it even can be used as a hydrophilic dihydroxy acid to endow PU with hydrophilicity. This can solve the issue of the lack of hydrophilic dihydroxy acid for WPU dispersions [91]. The bio-polyols from sunflower oil can also be prepared with high primary hydroxyl functionality by thiol-ene reaction [138]. Owing to the high content of hydroxyl polyols from sunflower oil, various kinds of chemical products, such as PU and polyester, can be synthesized and applied in a wide range with the assistance of ultraviolet curing [139,140,141]. Linseed oil can be modified via an esterification process, and is mainly employed as a chain extender for preparing WPU [142,143]. The obtained WPU presents good adhesion, durability and light resistance in wood coatings. Similar to the sunflower oil, the modified linseed oil can also be used as hydrophilic chain extender instead of dimethylol propionic acid (DMPA) for the preparation of anionic WPU [144]. Jatropha oil is modified to prepare polyol by epoxidation and oxirane ring opening reactions, with the advantages of low cost and easy extraction [145,146,147]. It can contribute to the colloidal stability and rheological properties for WPU, with various hydroxyl numbers [148,149]. Palm oil, consisting of triglycerides and diglycerides, can be modified to form an active polyol through epoxidation and transesterification processes for significant use in PU [150]. The modified polyol can be totally used to synthesize aliphatic PU and even PU foam with the absence of petrochemical-based polyol, indicating that the palm oil-based polyol has excellent reacting activity with isocyanates [151,152].

Generally, different kinds of PU products can be prepared by using the different bio-polyols from VOs, due to the abundant functional sites on the VOs chains (such as ester groups and C=C bonds). Apart from polyols, diols and active –OH terminal polymer can also be obtained to react with isocyanate for preparing PU, or to react with other fatty acids for preparing non-isocyanate PUs. Thus, the VOs can not only used to replace petroleum-based polyols, but also can be modified and employed for eliminating isocyanates for PU, which can supply a new path for producing bio-based PU.

#### 3.1.2. Cashew Nut Shell Liquid

**Figure 7:**
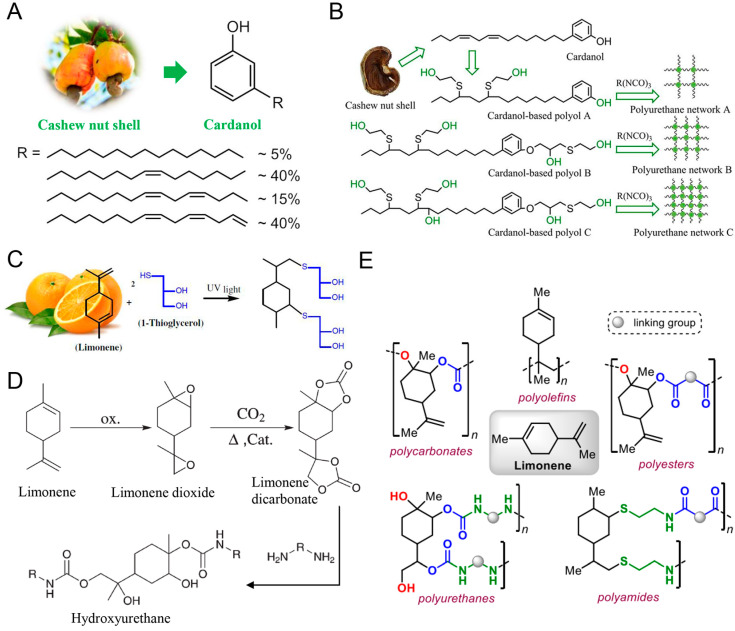
(**A**) Chemical structure of cardanol in cashew nut shell; (**B**) Polyols derived from cashew nut shells were used as the main building blocks of polyurethane. Reproduced with permission [156]. Copyright 2018, American Chemical Society. (**C**) Synthesis of limonene polyols by thiol-ene “click” chemistry. Reproduced with permission [162]. Copyright 2014, Springer. (**D**) Synthesis path from limonene to isocyanate polyurethane. Reproduced with permission [164]. Copyright 2012, Royal Society of Chemistry. (**E**) The application of limonene for different polymers. Reproduced with permission [157]. Copyright 2020, Science Press.

Cashew nut shell liquid (CNSL) is natural aromatic oil from cashew nuts, and can be used for the extraction of cardanol, which is a natural phenol containing a 15-carbon hydrocarbon chain in the middle and one, two or three non-conjugated double bonds, as depicted in Figure 7A [153,154,155]. Cardanol can be functionalized to prepare bio-polyols via esterification, epoxidation and hydrogenation processes, etc., and then reacted with isocyanates to synthesize PU (Figure 7B). The PU commonly presents good mechanical (compression strength of 471 kPa), thermal (higher than 250 °C) and fire properties. Wang et al. epoxidized cardanol to obtain polyol via a thiol-based click reaction [156]. The obtained polyol presents a high hydroxyl number, which can react with hexamethylene diisocyanate (HDI) trimers to synthesize thermosetting PU. The results show that the increasing number of hydroxyl groups in polyols has a positive effect on the crosslinking density of PU. In addition, the CNSL is able to be directly employed as polyol to prepare PU foams.

#### 3.1.3. Terpene

Terpene is a kind of natural hydrocarbons, which is widely found in limonene, pinene, myrcene, etc. [157]. It possesses a high reactivity due to the existence of relatively active C=C bonds in the various linear, cyclic and even polycyclic chains [158]. As one of the earliest used natural polymers, it derives two main functional polymers, including polyisoprene and polyolefins [159,160]. Among the derivatives of terpene, limonene can be the most important, which has been a commercial monoterpene from citrus fruits, as depicted in Figure 7C [161]. It can be facilely modified into bio-polyols through epoxidation or the thiol-ene click process under ultraviolet light (Figure 7C) or catalyst (Figure 7D), followed by the polymerization process to produce PU and NIPU, as well as other similar polymers, as illustrated in Figure 7E [157,162]. Bahr et al. prepared a terpene-based NIPU from cyclic limonene dicarbonate. Firstly, the limonene was epoxidized and followed by catalytic carbonation with CO_2_ to prepare cyclic limonene dicarbonate without ester groups. Then, the cyclic limonene dicarbonate was reacted with diamines in a non-isocyanate environment. The stiffness (Young’s modulus of 4100 MPa) and glass transition temperatures (62 °C) of NIPU might be enhanced significantly by increasing amine functionality, which reacts with cyclic limonene dicarbonate. It implies that the limonene dioxide can play a key role in synthesizing PU [163,164,165].

#### 3.1.4. Rosin

Rosin is a promising biomass for PU synthesis due to the merits of abundance, easy modification, and unique structure with cycloaliphatic, aromatic groups and hydrophenanthrene ring [166,167]. It is commonly used to prepare PU with excellent mechanical rigidity, heat resistance and adhesion [168]. Before the employment in PU, it is usually used directly or modified via isomerization and esterification processes [169,170]. Similar to the limonene, rosin can be also modified to prepare rosin-based cyclic carbonate, which is used to synthesize NIPU with amines [171]. This obviously broadens the raw materials for preparing NIPU in a green way. Notably, rosin is particularly added as a feedstock during the PU preparation process, which can endow PU with some interesting properties. It is reported that the heat resistance, viscoelasticity and antibacterial properties of PU can be increased by adding rosin. The PU presents an antibacterial rate above 99.2% against S.aureus [172]. Additionally, because the hydrophenanthrene ring of rosin is non-planar, it can supply a possibility of conformational transformation under the function of pressure. Thus, the rosin-based PU can possess excellent deformation and stretching strength [166,173]. The designed PU can present significant shape memory property at a high recovery rate. Zhang et al. reported this kind of PU by using a rosin-based diol as chain extender. It is found that the PU is good at shape memory, with an extensively strain higher than 1000% [174]. There is no doubt that these excellent mechanical and antibacterial properties can expand the application of PU in packaging areas.

### 3.2. PU from Lignin

**Figure 8:**
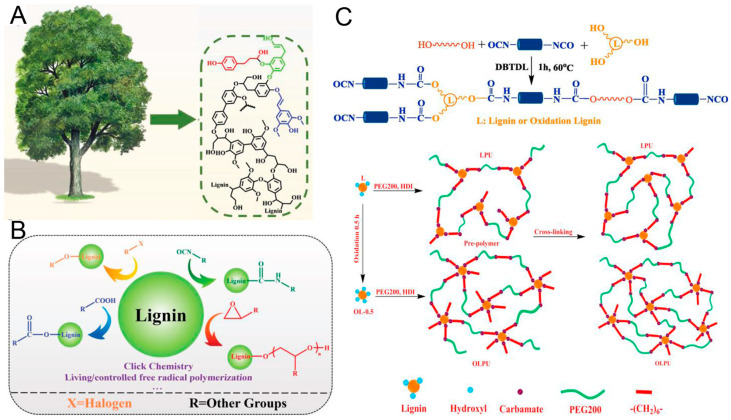
(**A**) The main alcohol structure in lignin; blue is octanol, green is coniferol, red is coumarol. (**B**) Common methods for lignin hydroxyl modification. Reproduced with permission [177]. Copyright 2020, Wiley. (**C**) Schematic diagram of lignin-based polyurethane synthesis reaction, internal structure diagram of LPU and OLPU. Reproduced with permission [194]. Copyright 2019, Elsevier.

There are a number of works of literature about the application of lignin and the derivatives in PU. Therefore, the lignin-based PU will be summarized briefly. As one of the most vital biomasses, lignin is easy to obtain from various resources, such as wood, grass, plant straw, etc. [175]. Unlike VOs, lignin and its derivatives do not compete with the food supply. Furthermore, there are abundant aromatic groups, phenolic and alcoholic hydroxyl, methoxy, and some other active groups [176]. As a comparison, lignin can be a better choice from an either resource- or from chemical structure-based perspective. As shown in Figure 8A, lignin mainly consists of the monomers of coniferyl, p-coumaryl and sinapyl alcohol. By changing the structures of monomers via oxygen radicals, lignin can present complex crosslinks with significant strength and hardness, as depicted in Figure 8B [177,178,179,180]. The modified lignin and the derivatives can be employed as polyol to prepare lignin-based PU [181,182]. Lignin can also indeed be added directly to PU during the polymerization process to supply multi-functional properties, such as antibacterial, oxidation and ultraviolet resistance, and even flame retardancy [183,184,185].

For the lignin from wood, the hydroxyl groups on lignin chains are able to react with isocyanates directly, under mild conditions, to obtain PU [186]. It is reported that lignin can be pre-treated via a solvent fractionation process to purify the complex heterogeneous structure and extract the homogeneous fractions of lignin [187,188]. Wang et al. investigated the function of softwood Kraft lignin fractions in lignin-based PU. The fractions were used as primary hydroxyl groups. It was shown that the rigidity and deformation resistance of PU could be enhanced by increasing the molecular weight of lignin [189]. Unfortunately, the obtained PU products are usually brittle and hard, by using lignin as the single polyol. To improve the viscoelasticity of PU, the other polyols are employed as secondary polyols, such as polyether, poly(ethylene glycol), and polyester, etc. [190,191]. This can provide a new path to design the mixture of lignin and synthesized polyols.

To improve the reactivity of lignin, it can be modified through various processes, including oxidation, esterification, isocyanate modification, oxypropylation, etc., as illustrated in Figure 8B. Among them, oxidation can be the most employed method to increase the alcoholic hydroxyl groups of lignin, as well as the functionality [192,193]. Zhang et al. mixed the ozone oxidized lignin with polyethylene glycol as the polyol mixture, to react with isocyanate for preparing PU (Figure 8C). It implies that the oxidized lignin presents a high hydroxyl content with good reactivity. The obtained oxidized lignin/polyethylene glycol PU shows a high degree of crosslinking with good performance [194]. Wang et al. purified lignin via co-solvent enhanced lignocellulosic fractionation pretreatment to obtain lignin-based products with narrow distribution and relatively low molecular weight. Then, the obtained lignin-based products were mixed with secondary hydroxyl groups (aliphatic diols and polyethers) to synthesize PU. It was found that secondary hydroxyl groups interacted with lignin molecules in different degrees, resulting in significant mechanical and thermal properties for PU [195]. Thus, lignin is surly a key candidate feedstock for PU, with unique characteristics [196,197,198]. Besides the roles of polyols, lignin can be used as fillers to modify PU elastomer.

Notably, lignin from plant straw shows different properties due to the lignocellulosic biomass from agricultural crops, including soybean [199], rape [200], wheat [201] and corn straws [202]. The lignin-based bio-polyols can be obtained by extracting products from these straws via a liquefaction process, which mainly consists of decomposition, esterification, and condensation polymerization processes [203]. Most of the liquefaction products are polyesters or polyether polyols with rich hydroxyl groups, indicating a good reactivity with isocyanates [204]. Hu et al. extracted bio-polyols from soybean straw by glycerol. The bio-polyols (hydroxyl numbers of 440–540 mg KOH g^−1^) are easily reacted with isocyanates to synthesize PU with good performance. The obtained PU can possess significant biodegradability [205,206]. It is suggested that the bio-polyols from plant straw can accomplish both the recycling of waste straws and the green synthesis of PU, which properly meets the needs of an environmentally friendly process.

With regard to the use of renewable resources and sustainable development, it is notable that there is a growing interest in the preparation of polymer materials using biomass as a raw material. The bio-based resource source is generally rich and the chemical structure is complex, with a large number of phenol hydroxyl and alcohol hydroxyl active groups. This allows for polycondensation or graft copolymerization chemical reactions. In addition to the extraction of a variety of valuable products, polyols and active -OH end polymers, which can be achieved through different means, can also be utilized in the synthesis of polyurethane. Some bio-based resource can also be directly involved in the synthesis of polyols, such as castor oil.

## 4. Biomacromolecules for Polyurethane

The synthesis of novel bioactive molecule-based polyurethanes has attracted more and more attention. Up till now, the most frequently used biomacromolecules are mainly polysaccharides and protein for PU synthesis and modification.

### 4.1. PU from Polysaccharides

Polysaccharides are carbohydrate polymers with a large molecular weight, and usually exist in the form of a main chain followed by side chain. Various carbohydrate polymers, including cellulose, starch, and cyclodextrin, etc., from plants, and chitosan from animals, have been used to prepare PU for wide applications in packaging, biomedicine, printing, coatings and adhesives, etc. [207,208,209]. Because of the branched structure and functional group of carbohydrates, it can be used as the composite material/filler in PU polymer matrices by covalent linkage with isocyanate to form a crosslinking network. Meanwhile, owing to the merits of renewable, non-toxicity, abundance and biodegradability, polysaccharides have been recommended as a promising candidate for preparing bio-PU with biodegradability [210].

#### 4.1.1. Cellulose for PU

Cellulose has been the most important, intriguing and abundant natural polymer, which consists of linear and long-chain glucose. It is abundant in hydroxyl groups, which supplies multiple points for possible H-bonding functions with other polymers [211,212]. According to the micro-morphology, the main cellulose products are cellulose fiber (micro- and nano-scale), cellulose nanocrystal (CNC), and bacterial cellulose (BC) [213,214], all of which have been devoted to WPU modification with excellent biodegradability, mechanical properties, and renewability [215,216,217]. Owing to the challenge of cellulose dissolving at a molecular level, it is hard to obtain a homogenous cellulose solution for polymerization during PU synthesis. Therefore, the different cellulose products are mainly used as fillers to modify PU, which are attested to be an effective way to improve the performance of PU [218,219,220].

**Figure 9:**
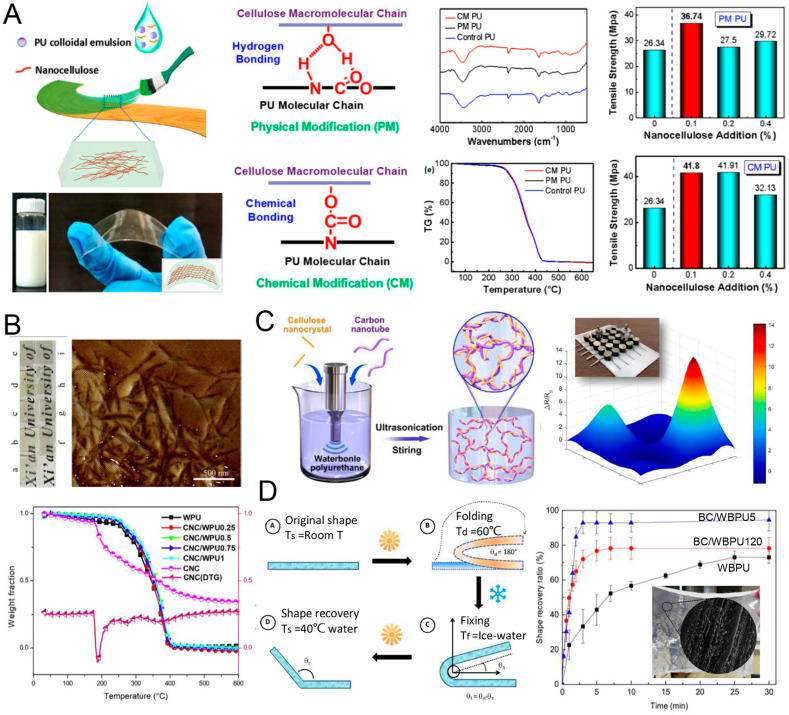
(**A**) WPU modified by cellulose nanofibers for application of wood coating; photograph of WPU, which contains 0.1 wt% of nanocelllose by chemical modification; photograph and schematic diagram of bending state of nanocellulose-modified polyurethane film; schematic diagram of hydrogen bond formation between PU macromolecule chain and nanocellulose (0.1 wt%) prepared by physical blending method, and the chemical bond by chemical modification method; the FTIR and TG curves of the two modified PU films; the tensile strength of physical modification of polyurethane (PM PU) and chemical modification of polyurethane (CM PU) with different nanocellulose content. Reproduced with permission [221]. Copyright 2019, MDPI. (**B**) AFM phase images and TG curves of WPU modified by cellulose nanocrystal. Reproduced with permission [223]. Copyright 2019, Elsevier. (**C**) Schematic illustration of WPU modified by cellulose nanocrystal/carbon nanotube application of piezoresistive sensors; a three-dimensional map of resistance and the inset is photograph of artificial electronic skin. Reproduced with permission [224]. Copyright 2020, Elsevier. (**D**) Fold-unfold shape memory test process and shape recovery curve in water, illustrated with wet film photograph and SEM image of cross-section 10,000. Reproduced with permission [229]. Copyright 2019, Elsevier.

It is reported that the cellulose nanofiber (CNF) can be introduced into WPU via the chemical grafting and physical blending processes, respectively. It is found that hydrogen bonding plays a key role to improve the performance of PU for physical blending, while a chemical bonding forms with PU chains for chemical grafting, as depicted in Figure 9A. Commonly, the chemical structure changes little, due to the insolubility of cellulose, and then the thermal stability is similar for PU and CNF-modified PU. Notably, CNF contributes to the tensile strength (~41.8 MPa, higher than the pure PU of ~26.3 MPa) of PU significantly [221].

As for CNC, it can be the most attractive biomass in nanoscales. It presents a rod-like nano-morphology with high crystallinity, which benefits the thermal and mechanical properties due to the hydrogen bonds’ interaction in nano-levels with PU chains. Our previous works have demonstrated this by using CNC from waste paper to reinforce WPU, as shown in Figure 9B [222,223]. CNC can also be modified further to endow WPU with novel properties, including electrical [224] and water resistance [225], antimicrobial properties, etc. [226]. In addition, CNC can also be mixed with VOs to modify WPU to present multi-functional properties, such as high hardness and compatibility, elastic recovery, and even good adhesion [227]. Zhang et al. combined CNC with carbon nanotubes as mixing fillers to modify WPU with high compressibility. This suggests that this composite can be used to prepare piezoresistive sensor with excellent sensitivity. It can hold great potential application in artificial electronic skin, as depicted in Figure 9C [224].

BC is a unique cellulose product from bacteria strains. It plays an important role in medicine, food and tissue engineering areas. Owing to the strong inter-chain hydrogen bonds, BC presents 3D nanofiber networks with significant mechanical properties in vivo. The unique structure and properties provide the possibility for applying BC as a hydrogel to cure wounds [228]. Urbina et al. prepared a gelatinous and translucent BC/WPU composite membrane with shape memory property. The shape memory property is adjusted by temperature, and the shape recovery ratio can be as high as 92.8 ± 6.3%. It is an excellent candidate for biomedical products, as illustrated in Figure 9D [229].

#### 4.1.2. Starch for PU

**Figure 10:**
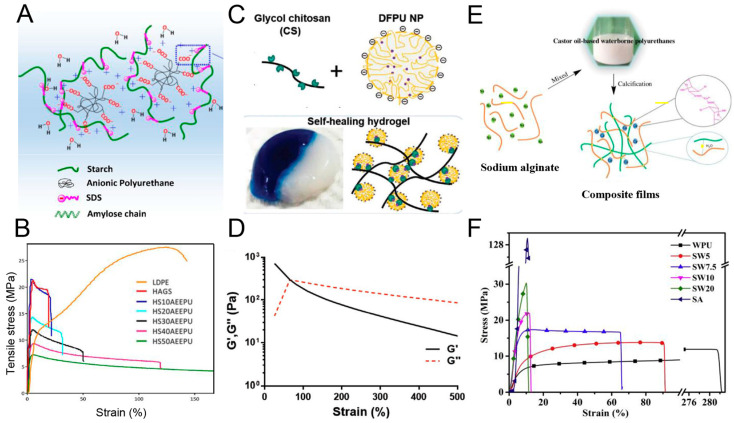
(**A**,**B**) Schematic diagram showing the physical crosslinking of starch and PU dispersion; stress–strain curves of HAGS (high amylose glycerol plasticized starch film), LDPE (low density polyethylene) and HAGS-AEEPU (Anionic polyurethane) films. Reproduced with permission [236]. Copyright 2018, Elsevier. (**C**,**D**) WPU modified by chitosan. Schematic representation of waterborne polyurethane with chitosan crosslinkers to form self-healing hydrogel or cryogel; the strain of self-healing hydrogel was up to 500%. Reproduced with permission [247]. Copyright 2020, Wiley. (**E**,**F**) Schematic diagram of process of sodium alginate (SA) modified WPU; stress–strain curves of PU films with different SA content. Reproduced with permission [250]. Copyright 2019, Elsevier.

Starch is not only a daily necessity in food for humans, but also the key natural polymer for chemical and material industries, due to the impressive physicochemical properties [230]. The introduction of starch into PU possesses significant superiorities, such as outstanding mechanical properties, elongation to break and modulus, biodegradability, which can extend the application ranges of PU in food, medicine and papermaking areas, etc. [231,232,233]. The hydrophilic nature of starch promotes the dispersion of it in WPU, as well as the preparation of NIPU composites [234,235]. However, one of the key problems is the micro-phase separation of physical incompatibility for starch/PU hybrids. It is suggested that the employment of a mixture of polyester (hydrophobic) and polyether polyol (hydrophilic) with starch can solve this problem. The prepared starch–polyurethane hybrid presents a good performance of elongation at break (187%), Young’s modulus (383 MPa) and contact angle (112°), and good transparency, as depicted in Figure 10A,B [236]. Similar to cellulose, starch also presents nanocrystals with a typical diameter (average equivalent diameter in the range 25–40 nm), which can strengthen the thermal stability and mechanical properties of WPU [210,237,238].

In addition, cyclodextrin is an enzyme produced from starch, and contains 6, 7 and 8 glucose units of cyclodextrin, which are called α-, β- and γ-cyclodextrin, respectively. Because it is composed of glucose, it has no toxic side effects, and can be absorbed by the body. Therefore, it is highly valued in the pharmaceutical field, used widely as a drug filler and binder, with the general properties of starch. Polysaccharide, as a multihydroxyl compound, can be conveniently used as a crosslinking agent in PU. Lee et al. introduced a β-cyclodextrin molecule, effectively migrated into the main chain of WPU via a sol–gel reaction to reinforce the thermal and mechanical properties, and biodegradability [239]. The β-cyclodextrin molecule consisted of d-glucopyranose groups linked by α-1,4-glycosidic bonds. Due to the presence of the external hydroxyl group of β-cyclodextrin, it has a hydrophobic inner cavity and a hydrophilic outer cavity. At the same time, it can be used as a multifunctional crosslinker to form chemical hybrids with organic polymers. Konieczny et al. synthesized stable PU films using the diisocyanate and an acetylated original, partially hydrolyzed amylopectin/white dextrin as a crosslinking agent [240].

#### 4.1.3. Chitosan for PU

Owing to the characteristics of non-toxicity, antibacterial, biological activity, cytocompatibility and degradability, chitosan have attracted huge attention, with great promise as biomaterials in both academic and industrial contexts [241,242]. There are active organic groups, including hydroxyl and amino groups, to support the chemical modification with other polymers, especially for PU [243,244,245,246]. For example, chitosan has been employed as a chain extender for preparing WPU with self-healing properties. The research found that the self-healing efficiency is about 47% for the tensile strength, which is much higher than that of WPU without chitosan (only 4%) [244]. This can be due to the exchange reaction between the hydroxyl group on the chitosan chain and the carbamate on the PU chain at high temperatures. Lin et al. used chitosan as a cross-linker to prepare biodegradable PU through a dynamic Schiff reaction at room temperature. As a result, the PU gels are sensitive to low pH values, with excellent self-healing properties of nearly 100% recovery after damage, as depicted in Figure 10C,D [247]. Furthermore, chitosan can be in situ incorporated in the form of nanoflakes into PU. It supports the PU with good shape recovery (~100% shape recovery ratio), an increase in tensile strength (~130%) and elongation at break (~500%). Meanwhile, the chitosan/PU composites also present a relatively high thermal stability of 360 °C. Therefore, chitosan can be introduced to PU synthesis either in chemical method or physically blending, resulting in different PU products. The chitosan supports PU to have great potential as biomedical materials [248].

#### 4.1.4. Sodium Alginate for PU

Sodium alginate is a kind of hydrophilic anionic polysaccharide, which is commonly used as seasoning in foodstuffs [249]. Owing to its water solubility, biological compatibility, and multiple physicochemical properties, it has been applied in biomedicine, packaging and printing, and even tissue engineering [250,251]. Wang et al. prepared sodium alginate/WPU composites by physically blending a sodium alginate aqueous solution with VO-based WPU. It is revealed that the hydrophobicity, mechanical properties, and thermal stability improve, and the elongation at break decreases by increasing the sodium alginate content in the composite. The changes of the composite properties can be caused by the improvement of crosslink density from the sodium alginate molecules with many active points, which provide the possibility for PU chains to form interpenetrating networks, as illustrated in Figure 10E. When the sodium alginate content in WPU increases from 5% to 20%, the tensile strength and Young’s modulus of the sodium alginate/WPU composite film gradually increases to the maximum of 26.5 MPa and 511 MPa (Figure 10F) [250].

#### 4.1.5. Glucomannan

Glucomannan, extracted from tubers, bulbs, cork, and roots, belongs to a kind of mannans family of polysaccharides, with structural and storage functions in plants [252]. It is mainly derived from konjac glucomannan, which is composed of β-1, 4 linked D-mannan and d-glucose monomers arranged in a block of mixed residues forming a linear chain [253]. The glucomannan can be successfully incorporated in PU to synthesize nanocomposites, while the synergistic effects of filler (glucomannan) and substrate (PU) provide outstanding mechanical properties due to the intermolecular hydrogen bond between PU and glucomannan [254]. It was found that PU and various types of glucomannan formed an interpenetrating polymer network-based graft material. The nitro derivatives of konjac glucomannan and castor oil-derived PU formed a semi-interpenetrating polymer network sheet by hydrogen bonding, which had good compatibility [253].

### 4.2. PU from Protein

Soy protein (SP) is a by-product of soybean oil industry. It is composed of amino acids, carboxylic acids, hydroxyl groups and mercaptan chemical functional groups. It is a kind of polymer, containing amino acids, which is considered to be a new raw material to replace polymers as petroleum resources to synthesize plastics [255]. Meanwhile, SP can be used as a filler in PU plastic, with many desirable properties, such as abundant resources, non-toxicity, low cost, biodegradability and renewability. Madbouly et al. prepared hydrolyzable PU/SP shape-memory polymer blends with improved crystallization behavior in environmentally friendly aqueous dispersions, which can be applied in multifunctional potential biomedical contexts [256]. Zhang et al. developed castor oil-based PU foam by a self-rising method, using water as foaming agent and SP as a reinforcing filler. The result showed that the crosslinking degree of the composite foams increased with the reaction of amino and hydroxyl groups on SP with polyisocyanates [255].

Not only plant proteins but also animal proteins can be used as raw materials for PU synthesis. A total of 90% of the feather structure is made up of a protein called feather keratin. Saucedo-Rivalcoba et al. showed that keratin is grafted into the PU structure by the amino and carboxyl groups of the amino acid structure to form polyurethane-keratin membranes. And beyond that, amino acids are the basic component of proteins, containing amino and carboxyl functional groups. It can also be involved in the synthesis of PU. Chan-Chan et al. reported the synthesis of more biocompatible and biodegradable PU, using unmodified amino acids as chain extenders via urea and amide formation, such as L-arginine, glycine or L-aspartic acid [257]. Among them, arginine and glycine have good effects on the properties of polyurethane materials, such as high molecular weight, deformation ability, tensile strength and high storage modulus. According to their good mechanical properties and biodegradability, PU prepared from amino acid has been extensively used in biomedical applications.

In general, the structure of biomacromolecule polysaccharides is complex and difficult to dissolve, which has led to their primary use in modifying polyurethane. This is achieved through chemical grafting and physical blending, which introduces the polysaccharides into the polyurethane chain and improves their mechanical and thermal properties.The protein is integrated into the polyurethane structure through the formation of covalent bonds between the amino and carboxyl groups of the amino acid structure, resulting in the synthesis of bio-based polyurethane products with biodegradability. As illustrated in Table 3, a diverse range of bio-based raw materials can be employed in the synthesis of polyurethane, resulting in a multitude of product types with distinct properties.

## 5. Sustainability for Polyurethane

Polyurethane is one of the most versatile materials in the world. It can be produced in a variety of forms and used in a variety of markets. According to the data, the polyurethane market was ranked sixth in global polymer production in 2018, with a total production of approximately 65.5 billion dollars. The market is expected to reach $105.2 billion by 2025 [258]. However, according to the data, recycled industrial waste and consumption waste polyurethane only accounts for 30% of the total content. Because the recycling of resources is largely driven by economic factors, cheap new materials recovered at the end of the life cycle are proving attractive. These products and processes must be further developed to make renewable technologies more competitive. Ultimately, the polyurethane feedstock minimizes dependence on non-renewable petrochemical products.

From an economic point of view, the total annual output of waste-based feedstocks and bio-based feedstocks and the cost of recycling these materials are shown in Table 4. We collected data and sorted out the total annual output of waste-based raw materials and bio-based raw materials and the cost of recycling these materials for a comparative analysis from an economic perspective. As can be seen from Table 4, waste-based raw materials generally have low recycling prices and relatively high annual yields. Due to the type and planting area of bio-based raw materials, the yield of some raw materials, such as CNSL, terpene, and rosin is low, so the price is higher. However, chitosan, cellulose and lignin are lower, due to the wide source and high yield. Generally speaking, the recycling of a single material does not require the removal of different plastics, greatly reducing the complexity of the process, and the homogeneous parts that can be easily removed from the end product all have a high recycling potential. There are many kinds of PU products produced from renewable recycled raw materials, including adhesives, elastomers, foams and coatings. At the same time, all kinds of PU products have excellent properties and can be widely used in various fields, such as cushion packaging, oil sorption, fireproof materials, biomedical implants.

In general, despite the recycling of resources to achieve the high value of the economic cycle, the industry is still facing many challenges. From the upstream design, collection and classification, to the last part of the regeneration process, the regeneration process is developing rapidly at present, but the real expansion of this market requires concerted action across the value chain. Upstream classification and sorting systems tend to be more efficient, and downstream access to high-quality raw materials will be less efficient.

**Table 4:** Recovery of different feedstocks of PU and their novel applications.

Feedstock	Yield (t/y)	Price ($/t)	Products	Applications	Ref.
PET	19.9 × 10^6^	418–448	PU Foams	Cushion packaging Oil sorption Fireproof materials	[42,43,259]
PU	12 × 10^6^	150–300	PU Foams	Foaming agent	[89,260]
PC	9.8 × 10^6^	700–900	PU films WPU	Biomimetic anti-fouling Coatings	[119,121]
VOs	90 × 10^6^	1800–2000	PU Foams	Mattress Ink	[3,261,262]
CNSL	0.03 × 10^6^	450–700	PU Foams coatings	Building materials Anticorrosive coatings	[153,263]
Terpene	0.45 × 10^6^	3000–3500	PU Foams	Flame retardancy	[264]
Rosin	0.65 × 10^6^	1800–2000	Elastomer	Shape memory material	[174]
Lignin	50 × 10^6^	50–200	Elastomer WPU	Anti-bacterial coatings Shoe Sole	[157,189]
Cellulose	100 × 10^6^	20–45	WPU Coatings	Wood antibacterial	[215]
Starch	30 × 10^6^	450–600	PU films	Biomedical materials	[239]
Chitosan	100 × 10^9^	2500–3000	PU gels Elastomer	Shape memory material Biomedical materials	[247,248]
Sodium alginate	0.23 × 10^6^	600–800	WPU	Biomedical materials	[252]
Glucomannan	0.25 × 10^6^	500–1000	WPU	Food packaging	[252]
Protein	0.2 × 10^6^	200–350	Elastomer	Biomedical materials	[256]

## 6. Conclusions and Prospects

Polyurethane is a kind of multipurpose resin widely used in many fields, in which one of main chemical composition is polyols. Apparently, the main resources of polyurethane are still petroleum feedstock. The scarcity of petroleum oil resources pulls the feed-stock of polyurethane toward recycled and renewable raw materials. These urgent requirements are driving research into more advanced polyurethane designs and have elevated effective approaches regarding the diversity of raw materials. To improve the environmental pollution problems, the two main ways summarized in this work are the solution, namely reusing waste petroleum-based polymers, and developing novel feedstock from natural resources. Therefore, the recent developments in PU preparation from waste, biomass and biomacromolecule resources have been reviewed concisely.

The PET, PU, and PC wastes are recovered and degraded into value-added polyols as the raw material to synthesize waste-based PU products, i.e., foams, films, coatings, and waterborne dispersion. Furthermore, the bio-based polyols are obtained from vegetable oil, cashew nut shell liquid, plant straw, terpene, lignin and some new biodegradable materials: polylactic acid and polycaprolactone. The bio-based resource sources, with a large number of phenol hydroxyl and alcohol hydroxyl active groups, are used to synthesize PU by polycondensation or graft copolymerization chemical reactions, and the bio-based PU products similarly have outstanding thermal stability and mechanical properties, applied to eco-friendly adhesive coatings and degradable packaging. Biomacromolecule polysaccharides are achieved through chemical grafting and physical blending, which introduces the polysaccharides into the PU chain and improves their mechanical and thermal properties. Meanwhile, the modification of PU is conducted by various biomacromolecules, such as polysaccharides and protein, to develop multi-purpose practical applications such as food packaging, biodegradable plastics and biomedical engineering. The utilization of recyclable and biomass materials has the effect of reducing the consumption of disposable mineral resources, such as petroleum, and the application of harmful chemicals. Furthermore, it provides a sustainable method of producing polyurethane. The incorporation of bio-based polyols renders polyurethane materials biodegradable, thereby mitigating the environmental contamination associated with PU products.

In a word, the use of waste-based recycled products or bio-based renewable raw materials in the production of PU not only satisfies the maximum utilization of resources, but also reduces the production costs for the circular economy principles. At the same time, it achieves the requirement of promoting green environmental protection and reducing pollution. Sustainable PU products, from recycling and renewable resources, have already gained huge attention for their promising application in printing and packaging, biomedical materials, buildings, and wearable electronics. While the future of PU, from the perspective of recycling and renewable resources, still holds great opportunity, it will also consist of tremendous challenges.

1. An important challenge for recycling resources is improved extraction, degradation and transformation, which are valuable building blocks that will be optimized at the cost of both the performance and monomers. At the same time, the purity and stability of the monomers prepared from recycling resources will also be an important consideration in the future research of PU.
2. From the upstream design, collection and classification, to the last part of the regeneration process, the regeneration process is developing rapidly at present, but the real expansion of this market requires concerted action across the value chain. Upstream classification and sorting systems tend to be more efficient, and downstream access to high-quality raw materials will be less efficient.
3. A crucial, and sometimes underestimated, synthesis route is the need to ensure the raw materials are compatible with current equipment condition. Although it is still early days to directly quantify and compare traditional petrochemical PU, there is enough research to show that recyclable materials have gradually become part of the view of the PU production, particularly regarding environmental pollution and fossil resource depletion.
4. Strengthen the connection between the solid waste classification and recovery system and the renewable resource recovery system, connect the renewable resource industry chain with the waste industry chain, improve the resource utilization rate, and strive to explore the industrialization development of the circular economy of polyurethane.

## Figures and Tables

**Table 1:** Comparison of different recycling methods of waste PET.

Methods	Degradation Reagents	Products	Potential Applications	Ref.
Glycolysis	Diethylene glycol Crude glycero Pentaerytheritol Trimethyloylpropane Ethylene glycol Neopentyl glycol Supercritical ethanol-ionic liquid	Polyol BHET DET Oligomer	PU foams for oil sorption PU adhesive Polyester-polyol PU coatings	[42,43,44,45,51,52,53,54]
Hydrolysis	Deionized water NaOH	TPA, EG	Aromatics Aromatic-derived compounds	[46]
Alcoholysis	Isooctyl alcohol Methanol	DMT EG	Polyvinyl chloride	[47,48]
Biodegradation	Sakaiensis bacterium Hydrolases	MHET, BHET TPA, EG	PU foams	[49,50]

**Table 2:** Comparison of different chemical recycling methods of waste PU.

Methods	Degradation Reagents	Products	Potential Applications	Refs.
Hydrolysis	CO_2_-water KOH-water	Polyol Diamines	Synthesis of PU Translated into isocyanate	[76,77,78]
Glycolysis	Diethylene glycol Ethylene glycol Glycerol Diglycerol Pentaerythritol Crude glycerine	Polyol Aromatic-carbamates	Synthesis of new flexible PU foam Thermoplastic PU and toluenediamines	[79,80,81,82,87]
Ammonolysis	Alkanolamines Dibutylamine ethanolamine	Polyol Aromatic amines Alkanolamine derivatives	Synthesis of new PUs melamine resins, epoxy resins, polyester, polycarbonates	[99,100]
Phosphorolysis	Phosphonic acids Phosphate Phosphoric acid Ethyl ester	Phosphorus Chlorine element oligomer	Flame retardant polyurethane or PVC materials	[104,105,106]
Hydroglycolysis	Water and glycols	Polyol Intermediate chemicals	Synthesis of new PUs	[107,108,109]

**Table 3:** The overview of the main bio-based feedstocks involved in molecular structure and properties of PU products.

Bio-Based Feedstocks	Molecular Structure	Products	Properties	Ref.
Vegetable oil	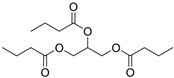	PU elastomer WPU	Mechanical properties Thermal resistance Physicomechanical stability Good adhesion	[128,129,134]
Cashew nut shell liquid	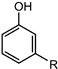	PU foams	Good mechanical, thermal and fire properties	[110,156]
Terpene	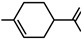	PU elastomer	Good mechanical properties	[161,162]
Rosin	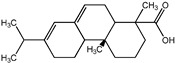	PU elastomer	Excellent mechanical rigidity, Heat resistance, shape memory property	[155,168,172]
Lignin	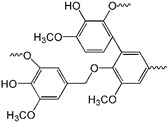	PU foams	Antibacterial, oxidation and ultraviolet resistance Flame retardant	[183,184,185]
Cellulose	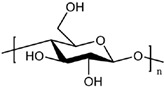	WPU	Biodegradability, mechanical properties, renewability Thermal properties	[221,229]
Starch	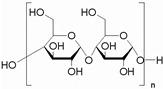	PU films	Good transparency, thermal properties Mechanical properties	[165,237,238]
Chitosan	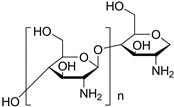	WPU PU films	Self-healing properties Good shape recovery	[197,247]
Sodium alginate	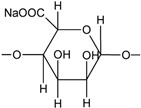	WPU	Hydrophobicity Mechanical properties Thermal stability	[250]
Glucomannan	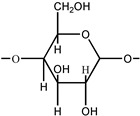	WPU	Outstanding mechanical properties Good compatibility	[253,254]
